# Multicolor single-analyzer high-energy-resolution XES spectrometer for simultaneous examination of different elements

**DOI:** 10.1107/S1600577522007561

**Published:** 2022-08-12

**Authors:** Antal Mikeházi, Jihad El Guettioui, István B. Földes, György Vankó, Zoltán Németh

**Affiliations:** a Wigner Research Centre for Physics, Konkoly Thege M. 29-33, 1121 Budapest, Hungary; ESRF – The European Synchrotron, France

**Keywords:** high-energy-resolution X-ray spectroscopies, von Hámos geometry, laser plasma, ray tracing, X-ray emission spectroscopy

## Abstract

The present work demonstrates the performance of a von Hámos high-energy-resolution X-ray spectrometer based on a non-conventional conical Si single-crystal analyzer. The analyzer is tested with different primary and secondary X-ray sources as well as a hard X-ray sensitive CCD camera. The spectrometer setup is also characterized with ray-tracing simulations. Both experimental and simulated results affirm that the conical spectrometer can efficiently detect and resolve the two pairs of two elements (Ni and Cu) *K*α X-ray emission spectroscopy (XES) peaks simultaneously, requiring a less than 2 cm-wide array on a single position-sensitive detector. The possible applications of this simple yet broad-energy-spectrum crystal spectrometer range from quickly adapting it as another probe for complex experiments at synchrotron beamlines to analyzing X-ray emission from plasma generated by ultrashort laser pulses at modern laser facilities.

## Abstract 2

This paper proves the feasibility and demonstrates the performance of a novel conical single-crystal analyzer in a von Hámos geometry to follow two sets of *K*α hard X-ray emission peaks simultaneously, which can be used as an additional feature at synchrotron beamlines but also with laboratory setups or plasma sources. The conical-analyzer-based spectrometer is studied both experimentally as well as with ray-tracing simulations to provide a broad overview and understanding.

## 1. Introduction

The evolution of high-energy-resolution X-ray spectrometers is still in progress not only at large-scale facilities, including synchrotrons, X-ray free-electron lasers (XFELs) and high-power laser facilities (see examples in Tu *et al.*, 2019 ▸; Khakhulin *et al.*, 2020 ▸; Zymaková *et al.*, 2021 ▸), but also in the laboratory, using X-ray tubes as sources (Németh *et al.*, 2016 ▸; Holden *et al.*, 2017 ▸; Malzer *et al.*, 2018 ▸; Błachucki *et al.*, 2019 ▸; Honkanen *et al.*, 2019 ▸). The most common devices, widespread at dedicated synchrotron beamlines, XFEL instruments, and recently even in laboratory setups, are Rowland-circle spectrometers equipped with a spherically bent crystal analyzer (SBCA) or von Hámos spectrometers (Hámos, 1939 ▸) using cylindrically bent analyzer crystals. They can both provide very high resolution X-ray spectra, although their working principles are different. In the Rowland-circle spectrometer the SBCA selects a single wavelength at a given Bragg angle, thus this device records intensity at a single energy at a time, and the spectrum is obtained by scanning the Bragg angle (while keeping the source, analyzer and detector on the Rowland circle) in the required range. The von Hámos spectrometer, on the other hand, is dispersive, and a position-sensitive detector can record an energy range limited often by the size of the detector, but large enough to cover the fine structure of an emission feature or an absorption edge. Despite their spread and success, these generalized spectrometer types do not cover all specific use cases, and alternative spectrometer designs with different analyzer geometries may emerge to be more advantageous. A frequent demand would be to simultaneously study the electronic structure of more than one element in a complex sample. Such a multicolor X-ray spectroscopy experiment is often not possible, as it requires two separate spectrometers including two separate analyzer crystals, often even two detectors (see, for example, Gul *et al.*, 2015 ▸). Even if it could be installed, the different experimental geometries required by the two spectrometers might not be possible to arrange due to limitations posed by beamline space restrictions, peculiarities of the sample environment, unfortunate positions of vacuum chamber openings, small windows of a high-pressure cell that require the two analyzer to be practically at the same position, *etc*. Thus a single spectrometer capable of recording both signals would be clearly highly advantageous.

As a viable alternative to routine X-ray emission spectroscopy (XES) setups, it was suggested by Hall (1984 ▸) that a conical-shape analyzer crystal can be used in the von Hámos arrangement, which results in a shorter spectrum with a more advantageous geometry. With such a device, not only is the spread of the spectrum squeezed tighter but the focal line is also rotated from the original one, that is parallel to the axis, to a perpendicular one, which is often more compatible with experimental setups. A conical von Hámos spectrometer was built for recording soft X-ray spectra using a KAP crystal (Martinolli *et al.*, 2004 ▸). Although the spectral resolution is somewhat lower and the alignment is more critical, it was successfully used in the soft X-ray range showing the satellite lines of the highly ionized aluminium (Martinolli *et al.*, 2004 ▸). Recently, multicone spectrometers have been designed (Bitter *et al.*, 2016 ▸) in order to improve the imaging properties and thus the spectral resolution with the applied X-ray streak camera.

A typical use case for such a distinct spectrometer is the study of shock-ignition-generated laser plasma via the measurement of the relative intensities of the X-ray emission signal of doped transition metal atoms in nano-structured targets. Here streak cameras with temporal resolution but limited spatial extent are used. Shock ignition (Betti *et al.*, 2007 ▸) is a promising way towards direct drive inertial fusion which separates target compression and ignition. Compression is achieved in a conventional way but with lower implosion velocity, using lasers with intensities of a few times 10^14^ W cm^−2^, which is followed by a high-intensity laser spike of about 10^16^ W cm^−2^ with some hundreds of picosecond duration launching a strong shock and igniting the fuel. In the case of shock ignition the hot electrons generated by the spike can be either beneficial (Shang *et al.*, 2017 ▸) or detrimental, depending on whether they are generated in Raman or two-plasmon decay instabilities. Therefore it is essential to characterize the hot electron temperature. The generally used method is X-ray spectroscopy. Emission of embedded radiators, *i.e.* microdots, contains *K*α radiation as electrons are kicked out of the *K*-shell by the fast electrons. Clearly, simultaneous time-dependent measurements by using two types of emitters at different depths of the target enable these investigations. Most of the current applications use two different high-resolution spectrometers for such observations (Cristoforetti *et al.*, 2017 ▸), but a single spectrometer which enables high-energy-resolution time-dependent observation of Ni and Cu *K*α radiation simultaneously would be more beneficial.

The aim of the present work is to demonstrate a conical von Hámos spectrometer built to observe hard X-ray emission lines around 8 keV to follow the changes of both Ni and Cu *K*α radiation simultaneously, and having the capability of time-dependent observation of Ni and/or Cu satellite lines that may appear when studying laser plasma with this spectrometer. Our choice for the analyzer crystal (and, correspondingly, the spectrometer geometry) was a bent Si(111) crystal (2*d* = 6.271 Å), manufactured by Saint Gobain (Saint-Gobain, 2021 ▸) by bending and gluing a 50 mm × 25 mm wafer to a conical surface made of aluminium, where the conical half angle of 14.78° was applied. The 50 mm dimension is in the dispersive direction and, orthogonal to this, the crystal is bent to a monotonously changing radius of curvature (from the conical axis) between its minimum of 95.68 mm and maximum of 108.44 mm at the edges, following the surface of a truncated cone. The spectrometer was designed to give an 800 mm source–detector distance (with the crystal in the middle, laterally 105 mm off from the source–detector center line). In this case the distance between the Ni and Cu *K*α lines is 17 mm in the detector plane, *i.e.* they can be well resolved either by a CCD or by an X-ray streak camera.

## 2. Ray-tracing simulations of the spectrometer

**Figure 1:**
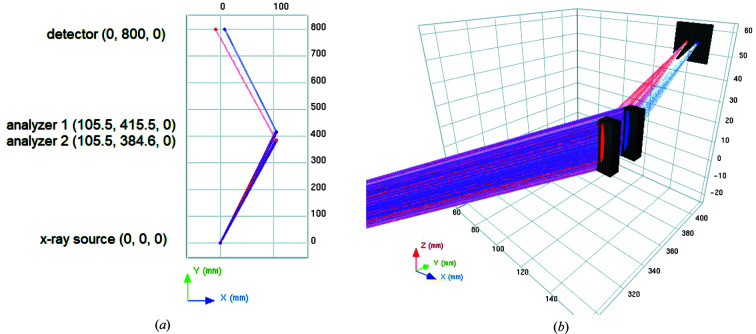
(Left) Layout of the *XRayTracer*-simulated spectrometer. The X-ray source is located at the origin; the two analyzer crystals, approximating the conical analyzer, are represented with the blue and red reflection points at *x* = 105.5 mm, while the detector plane is parallel to the *x*-axis and is at *y* = 800 mm (the center positions of the components as well as the scales are given in millimetres). (Right) 3D visualization of the setup used for the ray-tracing simulations. Red and blue tracks represent Ni and Cu *K*α radiation, respectively. The plasma source is not shown here; the detector surface is represented by the dark gray square at the far side of the picture, perpendicular to the line connecting the source and the detector. The conical analyzer is simulated by two narrow cylindrical analyzers with bending radius equal to that of the conical analyzer at the same position (109.7 mm and 103.4 mm for Ni and Cu, respectively).

In order to optimize the spectrometer setup, ray-tracing simulations can be beneficial. Fig. 1 ▸ shows the 3D visualization of the analyzer crystal and the detector surface of the intended spectrometer simulated by the *XRayTracer* software package (Klementiev & Chernikov, 2014 ▸). Although this software does not support conical-shape analyzers, we have used a model where two narrow (1 cm wide) cylindrical crystals with different bending radius (109.7 mm and 103.4 mm), according to the bending radius of the conical crystal at their position, mimic the conical surface. In the case of a conical von Hámos spectrometer, the Bragg angle and the corresponding position – where Ni and Cu *K*α signals are reflected – are separated on the crystal surface. Thus, in the simulations two cylindrical crystals were positioned at the calculated reflection segments of the Ni and Cu *K*α signals. This gives a good approximation of the actual conical analyzer crystal in the energy ranges of the Ni and Cu XES signals. The different bending radii at different X-ray energies are designed to make the focus of the signal on the detector perpendicular to the axis of revolution, as seen in Fig. 1 ▸. This is very beneficial at special environments like, for example, laser plasma chambers or sample vacuum chambers, which are often used at synchrotron beamlines.

**Figure 2:**
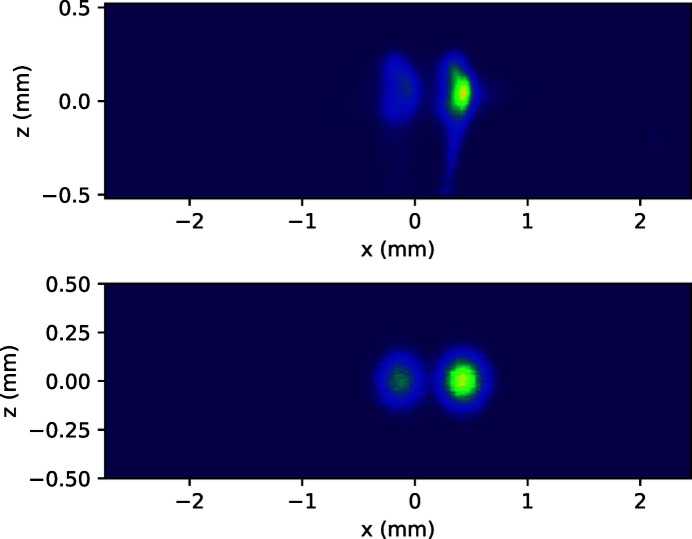
Experimental (top) and ray-traced (bottom) images of the conical analyzer reflected Cu *K*α emission lines.

**Figure 3:**
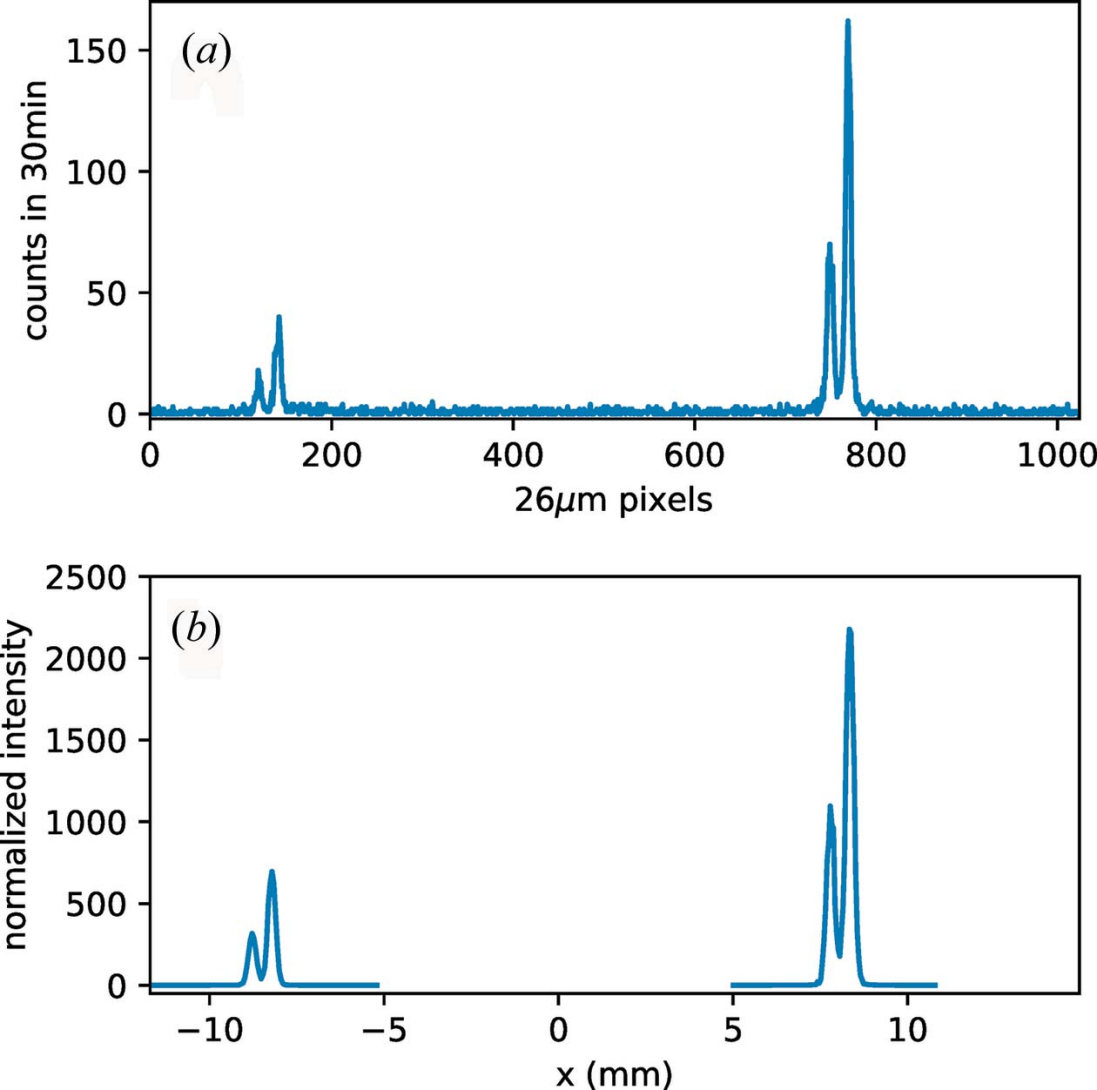
Ni (left) and Cu (right) *K*α peaks reflected from a 200 HUF coin onto the detector via the conical von Hámos crystal. Panel (*a*) shows experimental data, panel (*b*) the result of the ray tracing. Abscissas share the same scale, shown in pixel numbers in (*a*) and distances in millimetres in (*b*). The CCD detector has 26 µm × 26 µm pixels.

Fig. 2 ▸ shows the expected signal shape on the 2D detector (ray-tracing simulations on the bottom) compared with the measured ones (top) for the two Cu *K*α emission lines. The experimental setup includes the above-described conical Si(111) analyzer crystal and a position-sensitive 2D array hard X-ray CCD camera from Andor (Newton DY920, pixel size 26 µm × 26 µm). The primary X-ray source was an XOS XBeam X-ray tube with focusing optics, Rh anode and 50 W total power (the tube was set for 50 kV and 1 mA). The sample (secondary X-ray source) was the inner part of a 200 HUF coin (75% Cu and 25% Ni). The size and position of the ray-traced and experimental signals agree very well. When deducing the XES signals from the detector image, a good agreement is found between ray-tracing results and measurements for both the Ni and Cu emission peak pairs (Fig. 3 ▸).

## 3. Energy- *versus* wavelength-resolved X-ray spectra

In the case of X-ray fluorescence (XRF) spectroscopy, where multiple emission X-ray peaks from different elements have to be recorded simultaneously, commercially available energy-resolving solid state detectors are used. For example, a silicon drift diode (SDD) provides sufficient energy resolution to quantitatively determine elemental ratios. However, both the energy resolution and the temporal resolution of these semiconductor detectors are limited, constraining their use in some cases, like characterizing laser-induced plasma, as described in the *Introduction* [Sec sec1].

**Figure 4:**
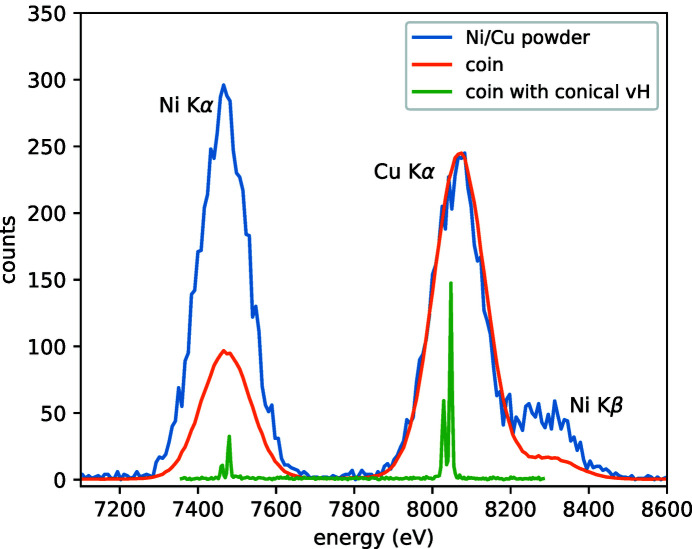
Comparison of *K*α emission signals recorded by an energy-resolving solid state detector (Amptek SDD; mixed metallic powders: blue curve; inner part of a 200 HUF coin: orange) as well as a wavelength-resolving conical von Hámos spectrometer (200 HUF coin inner part: green).

The difference in energy resolution between an energy-resolving solid state detector and a wavelength-dispersive crystal spectrometer can be easily demonstrated with laboratory X-ray sources. An example is given in Fig. 4 ▸, where hard X-ray fluorescent signals from metallic Ni and Cu both in a 1:1 ratio powder mixture as well as in a metallic alloy (inner part of a 200 HUF coin, Cu:Ni ratio 3:1) recorded by an Amptek SDD X-123 unit (∼25 cm away from the sample and with 45° angle to the line of X-ray propagation) and a wavelength-dispersive von Hámos spectrometer (setup as described in Section 2) are compared. While the overall efficiency is superior when using a single SDD detector, the two orders of magnitude better energy resolution of the von Hámos spectrometer is also obvious.

## 4. Optical characterization of the conical analyzer crystal

**Figure 5:**
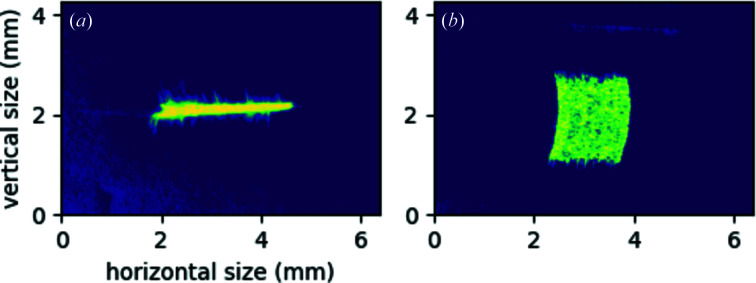
(*a*) Optical image of a diffuse light source reflected from the conical analyzer in horizontal von Hámos geometry (see text for details), the detector being in the focus. Note that the image does not represent real colors, only intensity. (*b*) The same image as for (*a*), but the detector is moved closer to the source.

To image the anticipated reflection area from the conical analyzer to the detector plate in the aforementioned von Hámos geometry, a diffuse green laser source was deployed in the source position. The Si crystal reflected light gives a good approximation of the total area covered by the conical crystal on the detector. Fig 5 ▸ demonstrates two cases: panel (*a*) shows the reflected optical image on the optimal detector position, while panel (*b*) does the same for a surface which is much closer to the source. The former results in a 2.6 mm-wide (dispersive direction) well focused line (focus height is about 0.2 mm). In contrast, if the detector plate is not in the optimal position, the focus is greatly distorted [in the case of Fig. 5 ▸(*b*), 1.7 mm high], while the width of the image is thinner.

## 5. X-ray mapping of the surface of the conical crystal

**Figure 6:**
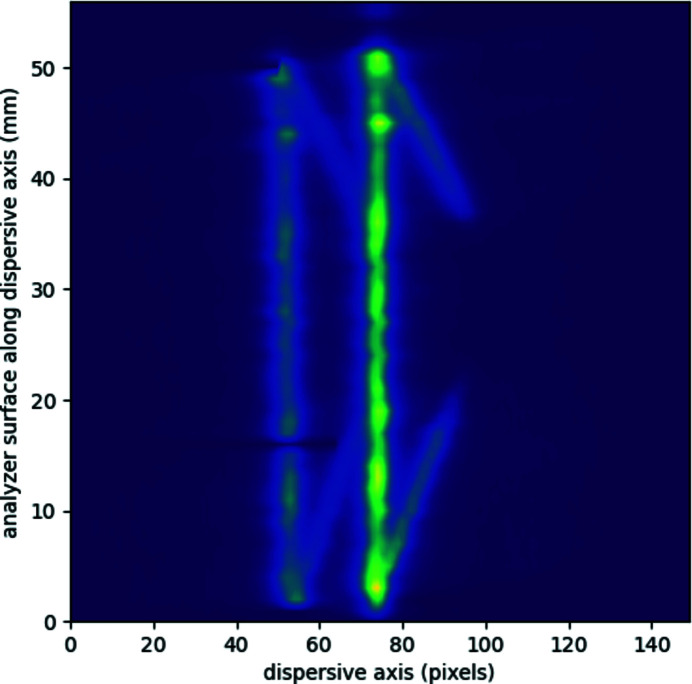
Reflection map of the conical analyzer crystal. The integrated energy spectrum on the detector is plotted horizontally, the maximum of the *K*α signal is aligned to pixel 74. The crystal surface is scanned with the direct beam of the X-ray source anode (mostly the Cu *K*α double peak). The vertical axis shows the relative shift of the analyzer crystal along its nodal line (thus it represents the segment of the crystal where the Cu *K*α peaks are reflected). The diverging peak pairs from both bottom and top show an extra reflection from the edge of the analyzer (see text).

An important note on the specific conical shape analyzer crystal used in this work is that during the first test measurements unexpected peaks with varying intensity and position appeared near the Cu and Ni *K*α peaks. A full scan of the crystal revealed the nature of this anomaly. For this purpose, the direct X-ray beam from another XOS XBeam microfocus tube (here with Cu anode) was used as source, pointing towards the analyzer. The analyzer crystal and the CCD detector were aligned in the aforementioned von Hámos geometry, but considering the focal point of the polycapillary optic being the origin to record the Cu *K*α peaks of the tube anode. The spectrometer (the analyzer and the detector) was shifted along the dispersive direction of the analyzer (axis *y* in Fig. 1 ▸) to scan the crystal surface with the beam, thus using different segments of the crystal to reflect the Cu *K*α radiation. The resulting intensity map is shown in Fig. 6 ▸. Each row in the map corresponds to the (in the non-dispersive direction) integrated signal of the CCD detector (that is, the resolved energy spectrum), while the vertical axis refers to the position of the X-ray beam center on the 50 mm-long conical crystal. The spectra are aligned to have the *K*α signal global maxima at pixel 74 in the dispersive axis, compensating the shift of the signal on the detector surface due to the different bending radius at different positions on the conical analyzer.

**Figure 7:**
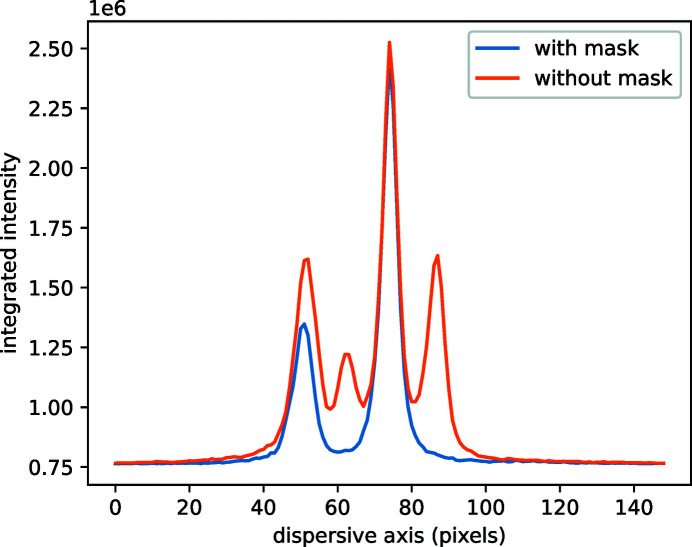
Horizontal cut of Fig. 6 ▸ at 13 mm on the ordinate (orange curve). Blue data show the same acquisition with masking a 1 mm-wide segment of the crystal at its edge with a Pb foil.

Normally, one would expect only two intensity maxima (Cu *K*α_1_ and *K*α_2_) with intensity ratios of 2:1 in each row over the entire map, but additional signals deviating from both peaks and both at the bottom as well as at the top can be clearly identified. One exemplary row is plotted in Fig. 7 ▸ as the orange line, the extra lines appearing at about pixels 62 and 87. However, if the very edge of the crystal is masked out with lead (about 1 mm from both left and right sides), the extra reflections disappear completely, as shown in Fig. 7 ▸ by the blue line. Thus, the extra peaks arise due to a reflection of the diverging radiation from the edges of the analyzer crystal.

## 6. Alignment robustness

In the proposed example application of the conical-based von Hámos spectrometer, that is, measuring hot electrons generated in laser plasmas, the source position (the focal point of the laser) is considered to be well defined, but the positioning of the analyzer and, more probably, the detector can be restricted by spatial constrains (*e.g.* by the fixed dimensions of the vacuum chamber). While the alignment of the analyzer is usually critical in the von Hámos geometry, the positioning of the detector is considered to be more flexible. In order to test the robustness of the detector’s position in the conical-analyzer-based XES spectrometer, the *K*α XES spectrum of the Cu anode of an X-ray tube (XOS XBeam) was measured with the above-described conical analyzer and Andor CCD detector with optimized crystal alignment but shifting the detector around the optimal position by a few centimetres along the axis of revolution of the analyzer (axis *y* in Fig. 1 ▸).

**Figure 8:**
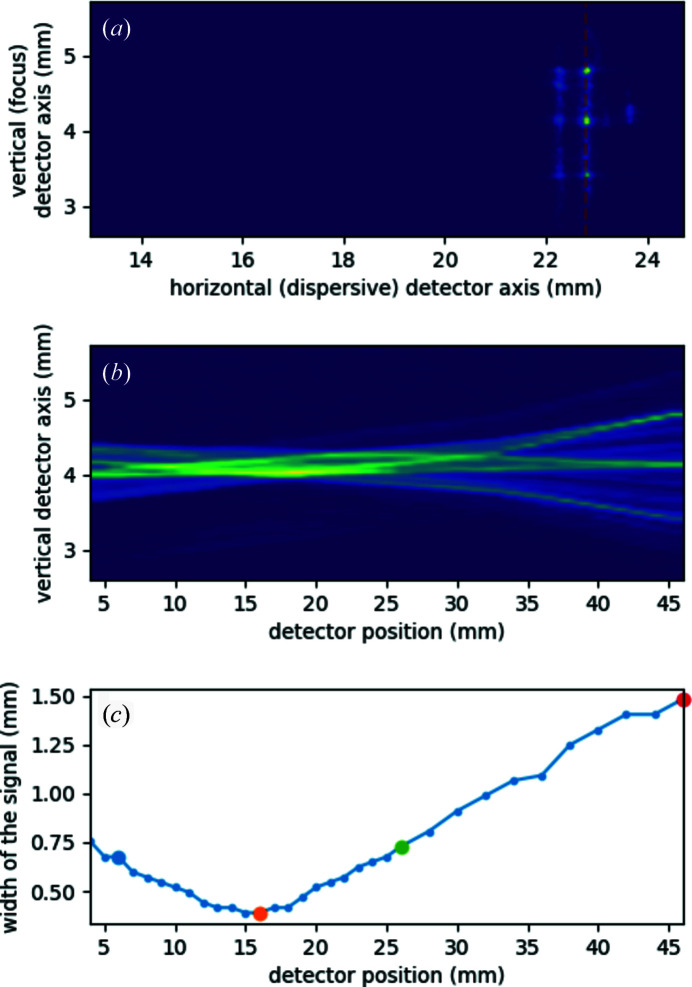
(*a*) Cut of the total raw detector image recording the Cu *K*α XES spectrum of a polycapillary Cu anode X-ray tube via the conical-analyzer-based von Hámos spectrometer. The horizontal axis corresponds to the dispersive direction of the spectrometer, the vertical axis to the focusing direction. Intensity units are untreated ADU. The X-ray tube was operated at 50 kV and 1 mA, acquisition time was 10 s. (*b*) Effect of sliding the detector along the line connecting the detector and the source (‘detector position’ axis) on the focal size of the *K*α_1_ peak. The latter is extracted from each single detector image by choosing the column with the highest intensity [an example of the chosen data column is represented by the orange line in (*a*)]. (*c*) Width of the focus of the chosen *K*α_1_ peak column as a function of detector position.

A typical spectrum image in a non-optimal detector position is shown in Fig. 8 ▸(*a*). The *K*α_2_ and *K*α_1_ lines appear as thin and high signals around nominal 22.5 and 23 mm, well separated from each other. The focus (vertical extension) is rather wide, almost 1.5 mm, and the signal is structured vertically to dominating dots. The latter comes from the fact that the source is a polycapillary focused X-ray tube, which emits a non-homogeneous, dotted cross-section X-ray spot due to the small capillaries, thus the emission line is not a spatially homogeneous line, as well. While changing the detector position, the *K*α peaks retain their shape in the dispersive direction, but the focus varies greatly. This is shown in Fig. 8 ▸(*b*), where the vertical line with the strongest intensity of all detector images [*e.g.* the orange line in Fig. 8 ▸(*a*)] is plotted as a function of nominal detector position. The focal height of the individual *K*α_1_ lines are also plotted in Fig. 8 ▸(*c*). It is clearly seen that the optimal focus is retained for about 5 mm (around nominal 16 mm), but, even at 3 cm off, the focus is only 1 mm wider.

**Figure 9:**
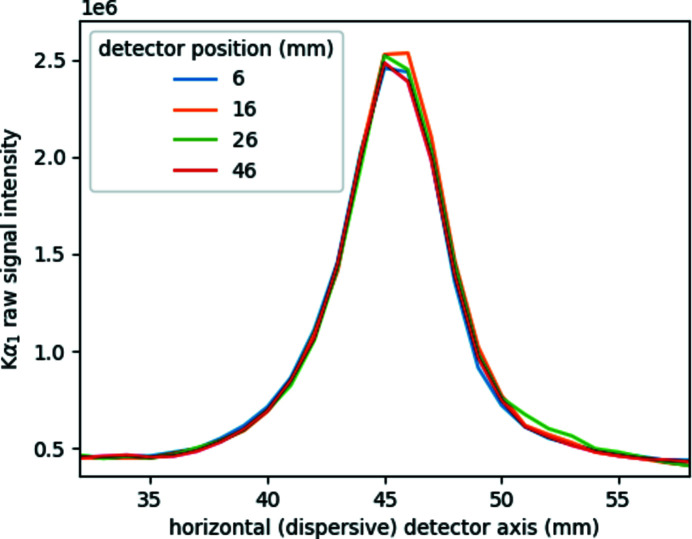
Cu *K*α_1_ peaks recorded by the conical-based von Hámos spectrometer with a Cu anode polycapillary source at different detector positions (see text and caption of Fig. 8 ▸).

In contrast to the changes in the focal size, the spectral shape, that is, the energy resolution of the spectra, remains unperturbed by the mispositioning of the detector. This is illustrated in Fig. 9 ▸, where the *K*α_1_ peak is plotted at four different detector positions. It is recognizable that the spectra are identical and the detector positioning does not affect the line shape.

## 7. Conical *versus* cylindrical crystal analyzers

**Figure 10:**
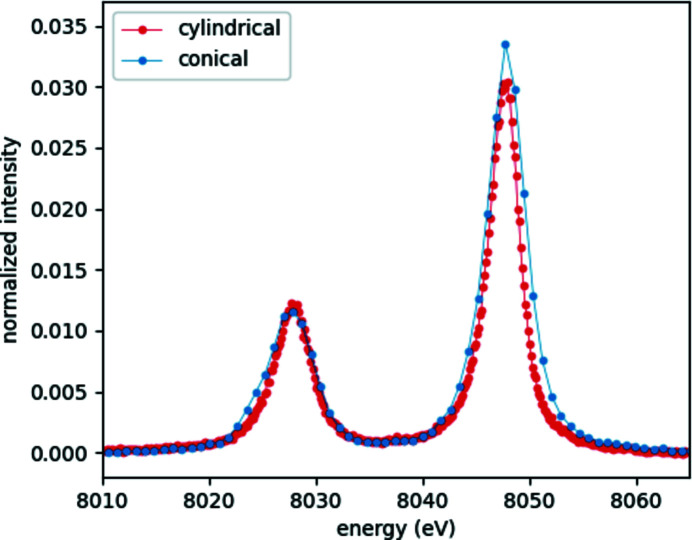
Cu *K*α spectrum of a metallic Cu foil recorded by von Hámos spectrometers with cylindrical (red) and conical (blue) analyzer crystals.

While the advantages of the von Hámos spectrometer with the conical analyzer crystal over the energy-resolving solid state detectors are obvious, it is worth comparing this setup with the more widespread cylindrically bent analyzer-based von Hámos spectrometers. The latter uses a simpler, thus cheaper, crystal which focuses the dispersed radiation on a line along its axis of revolution, twice as long as the footprint of the diffracting surface of the analyzer crystal (Hámos, 1939 ▸; Hoszowska *et al.*, 1996 ▸; Szlachetko *et al.*, 2012 ▸). This results in a relatively narrow observable energy range as well as high sampling rate of the high-energy-resolved spectrum. In contrast, the conical analyzer reflects a spectrum with larger energy range and lower sampling. To test these effects, two spectrometer setups are compared here. The conical-analyzer-based one is very similar to what was used in the preceding sections, the only difference being the detector, which was a Dectris Mythen2 1D strip detector with 50 µm-wide strips here. The cylindrical spectrometer uses a segmented Si(531) crystal (100 × 50 mm total area with 100 pieces of 1 mm-wide, 50 mm-long strips) with 250 mm bending radius, the geometry corresponds to the mean Bragg angles of either the Cu or the Ni *K*α peaks. The detector is the same Mythen2 strip detector, as used with the conical setup. Fig. 10 ▸ gives a comparison of the observed spectra from a Cu foil secondary source (excited by an XOS XBeam X-ray tube with Rh anode and polycapillary optics) for the two spectrometers, in good agreement with the above-described considerations. It is worth mentioning that the Si(531) crystal has a slightly better energy resolution for the measured Cu XES signal due to the different Bragg angles applied. The aim of this comparison is to contrast the spectra of the present geometry-restrained conical spectrometer with an almost optimal setup based on the conventional cylindrical von Hámos spectrometer.

**Figure 11:**
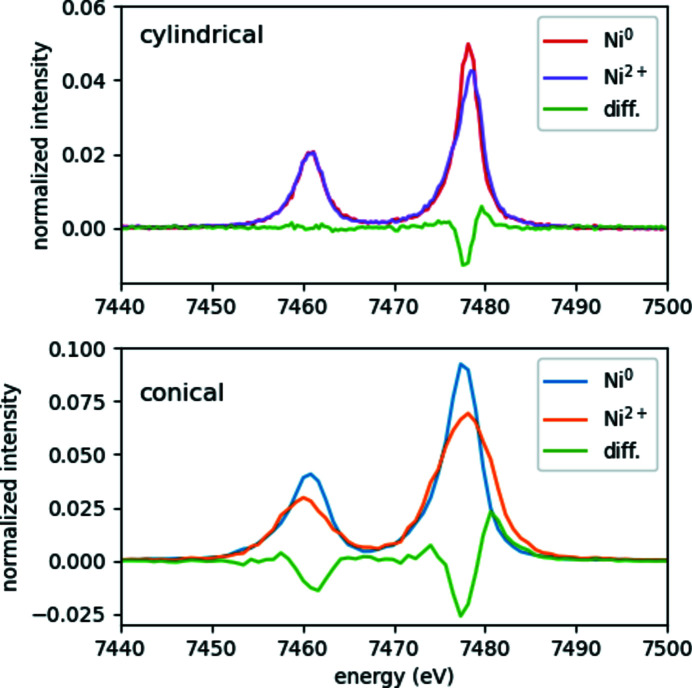
Ni *K*α spectra of metallic Ni and ionic Ni^2+^ (in the form of NiSO_4_ powder) recorded by von Hámos spectrometers with cylindrical (top panel) and conical (bottom panel) analyzer crystals.

As foreseen, the conical-analyzer-based setup can record both Ni and Cu XES, while the cylindrical based one can measure only one element with a given setting. In terms of efficiency, both the cylindrical as well as the conical-analyzer-based spectrometers resulted in very similar count rates integrated for the two *K*α peaks for both elements investigated. While the data from the conical-based setup is sufficient to analyze peak intensity ratios, both the lower sampling as well as the setup’s high sensitivity to precise alignment makes it less favorable for line-shape-analysis-based evaluation, *e.g.* for measuring electronic or spin structure differences in different chemical environments. This is illustrated in Fig. 11 ▸, where the *K*α spectra of metallic and ionic (divalent) Ni atoms recorded with conical- and cylindrical-analyzer-based spectrometers are shown. While both data sets show pronounced differences between Ni^0^ and Ni^2+^, the lines registered by the conical setup are much broader and the different broadening due to small misalignment during sample changing hinders the signal-shape analysis significantly.

## 8. Conclusions

The applicability of a conically bent Si crystal analyzer in a von Hámos type dispersive spectrometer to record Ni and Cu *K*α XES signals simultaneously is presented. The conical-analyzer-based spectrometer works with good efficiency and adequate energy resolution and is suggested to be well applicable to, for example, synchrotron beamlines as an alternative option to the existing single-element spectrometers or in laser-plasma experiments, especially for those intending to measure the hot electron temperature, related to shock ignition inertial confinement fusion.
